# When nerves hit: The effect of trait anxiety, situational stress, and task mastery on the perception and interpersonal accuracy of musical expressiveness

**DOI:** 10.3389/fpsyg.2022.1061922

**Published:** 2023-01-11

**Authors:** Álvaro M. Chang-Arana, Anastasios Mavrolampados, Niklas Pokki, Marc R. Thompson

**Affiliations:** ^1^Brain and Mind Laboratory, Department of Neuroscience and Biomedical Engineering, Aalto University, Espoo, Finland; ^2^Department of Music, Art and Culture (MACS), University of Jyväskylä, Jyväskylä, Finland; ^3^Department of Piano, University of Arts Helsinki – Sibelius Academy, Helsinki, Finland

**Keywords:** music performance anxiety, trait anxiety, situational stress, task mastery, interpersonal accuracy

## Abstract

Music performance anxiety (MPA) is a prevalent phenomenon with potentially serious consequences to a musician’s wellbeing and professional career. Yet, MPA does not always affect performance quality. It is hypothesized that trait anxiety, situational stress, and task mastery can exacerbate the effects of MPA and affect performance quality. Furthermore, it is unclear whether these effects are noticeable to both listeners and performing musicians. We measure performance quality as the expressiveness scores assigned by musicians and listeners to a set of pre-recorded performances. We selected three pianists with low, mid, and high MPA. Each pianist performed two pieces of their choice, familiar and unfamiliar, which were performed in rehearsal and recital conditions. The performances were videoed and edited into shorter clips for being presented to the performing pianists and to a set of online raters. Listeners and pianists will be asked to rate the expressiveness of all clips. We will determine the difference between the listeners’ perceived expressiveness and the pianists’ own expressiveness scores to estimate how well did listeners understand the pianists’ expressive intentions. We investigate (1) what is the effect of trait anxiety, situational stress, and task mastery on the listener’s perception of expressiveness and (2) what is the effect of these same variables on the listeners’ understanding of expressiveness.

## 1. Introduction

Music performance anxiety (MPA) is a prevalent phenomenon (Fernholz et al., 2019) among musicians, affecting them from early to post stages of a performance (Kenny, 2011; Chang-Arana et al., 2022). On its more severe manifestations, a musician may develop mood disorders (Kenny, 2011), choose to quit an otherwise promising career (Hernández et al., 2018; Fernholz et al., 2019), and engage in unhealthy strategies to cope with its debilitating symptoms such as drug consumption (Taylor and Wasley, 2004; West, 2004; Brugueìs, 2011a,b; Hernández et al., 2018). Kenny (2010) defines MPA as:

The experience of marked and persistent anxious apprehension related to musical performance that has arisen through specific anxiety-conditioning experiences. It is manifested through combinations of affective, cognitive, somatic, and behavioral symptoms and may occur in a range of performance settings, but is usually more severe in settings involving high ego investment and evaluative threat. It may be focal (i.e., focused only on music performance), or occur comorbidly with other anxiety disorders, in particular social phobia. It affects musicians across the lifespan and is at least partially independent of years of training, practice, and level of musical accomplishment. It may or may not impair the quality of the musical performance (p. 433).

Despite the problems associated with MPA, it does not always affect performance quality (Kenny, 2011; Osborne et al., 2014). Under what circumstances can MPA affect performance? According to Wilson and Roland (2002) and others (Papageorgi et al., 2007; Matei and Ginsborg, 2017), there are three sources of stress in the context of music performance which can exacerbate the effects of MPA and affect performance quality: trait anxiety, situational stress, and task mastery.

Trait anxiety is “any personality characteristics, constitutional or learned, that mediate susceptibility to stress” (Wilson and Roland, 2002, p. 50). More specifically, Spielberger et al. (1983) defined it as the “differences between people in the tendency to perceive stressful situation as dangerous or threatening and to respond to such situations with elevations in the intensity of their state anxiety (S-Anxiety) reactions” (p. 5). A brief distinction between stress and anxiety is needed. While stress and anxiety share nearly identical symptoms, the former refers to emotional reactions in response to external triggers; while the latter refers to persistent emotional reactions even in the absence of such triggers (American Psychological Association, 2022). Some evidence suggests that trait anxiety and MPA are strongly correlated (e.g., Chang-Arana et al., 2018, reported an *r* = 0.70). That is, there is a large overlap between both concepts. Yet, while trait anxiety refers to overall tendencies to assess situations as threatening, MPA circumscribes the threat assessment to the musical context and takes into account the particularities and challenges specific to music performance.

Situational stress is “environmental pressures such as public performance, audition, or competition” (Wilson and Roland, 2002, p. 50). Similarly, Papageorgi et al. (2007) claimed that the “presence of an audience, the amount of perceived self-exposure and venue characteristics are considered to be significant variables” (p. 91). That is, MPA may manifest strongly in contexts where there is a sense of higher ego investment (Kenny, 2011). As we have reviewed earlier (Chang-Arana et al., 2022), past experimental studies in MPA suggest that musicians experience higher MPA (manifested through self-report and physiological manifestations) and lower performance quality scores when performing in front of an audience vs. when playing alone (Brotons, 1994; LeBlanc et al., 1997; Yoshie et al., 2008, 2009; Wells et al., 2012; Kwan, 2016). Although in our previous work (Chang-Arana et al., 2022) we did not find an effect of performance context on the listener’s perception of MPA.

Task mastery ranges “from performances of simple, well-rehearsed works to those of complex, unprepared material” (Wilson and Roland, 2002, p. 50). Studies indicate that tertiary music students (Kenny et al., 2011; Casanova et al., 2018), as well as professional musicians (Roland, 1994; Kenny et al., 2012; Biasutti and Concina, 2014) report inadequate preparation for a performance as a cause for experiencing MPA. Conversely, higher self-efficacy in tertiary music students relates to less self-reported performance anxiety (Zarza-Alzugaray et al., 2016a). Yet, to the best of our knowledge no experimental studies in MPA have tested the effect of unprepared performances on the musician’s experience of anxiety and the listener’s perception of performance quality.

One indicator of performance quality is musical expressiveness (Thompson and Williamon, 2003; Wapnick et al., 2004; Kwan, 2016). Musical expressiveness has been defined as “those aspects of a musical performance that are under the control of the performer, and which the performer manipulates for aesthetic and communicative purposes. These may be considered aspects of musical prosody (Bernstein, 1976/1981)” (Bhatara et al., 2011, p. 921). Acoustically, expressiveness is a complex construct associated with variations in timing, dynamics, timbre, articulation, and intonation occurring during the interpretation of a piece (Davidson, 1993; Broughton and Stevens, 2009; Thompson and Luck, 2012; Vuoskoski et al., 2014).

Some studies have investigated how the listener’s perception of different performance quality metrics, including expressiveness, change according to the listener’s musical background (Stanley et al., 2002; Wapnick et al., 2004; Thompson, 2006; Geringer and Johnson, 2007; Johnson and Geringer, 2007; Broughton and Stevens, 2009; Broughton and Davidson, 2014). Musicians can detect differences in performance quality of ensembles of different musical level (Geringer and Johnson, 2007; Johnson and Geringer, 2007). The skills to discriminate performance quality may also depend on the listener’s main instrument (Wapnick et al., 2004; Broughton and Davidson, 2014). Kwan (2016) reported differences in the listener’s perception of expressiveness and performance quality, depending on their musical background. Our own research (Chang-Arana et al., 2022) suggests that musicians perceive more anxiety in a technically challenging piece when compared to non-musicians.

Musicians aim at communicating their expressive intentions to the listeners (Spiro and Schober, 2021) and endure uncountable hours of practice, as well as emotional, physical, and professional pressures to do so (Czerwiński et al., 2022). Yet, rarely do we know whether listeners are capable of perceiving accurately the performer’s expressive intentions. Such a comparison would allow evaluating the effectiveness of communication between musicians and listeners, providing musicians with a source of information of what listeners understand from the performances they listen to.

The concept of interpersonal accuracy allows investigating whether listeners perceive accurately the musician’s expressive intentions. Interpersonal accuracy is the “accurate judgment about any verifiable characteristic of a person or about the group that a person belongs to” (Hall et al., 2016, p. 5). The notion of accuracy is always abstract and context-dependent; thus, it is necessary to operationalize what constitutes an accurate judgment in a specific research context (Hall et al., 2016). In the context of this study, the listeners’ interpersonal accuracy is defined as the difference between the listener’s perception of expressiveness and the pianist’s self-reported expressiveness; the lower the difference, the higher the listener’s interpersonal accuracy (Chang-Arana et al., 2022).

Someone’s interpersonal accuracy (IA) is influenced by different contextual factors (Schmid, 2016). These can be as diverse as belonging to a particular socioeconomic status (Bänziger et al., 2011; Bjornsdottir et al., 2017), adopting similar body postures with the interacting partner (Fujiwara and Daibo, 2022), and even being in a violent relationship (Clements et al., 2007). The experience from previous studies suggests that the listeners’ skills to accurately infer the expressive intentions of a performer may be influenced by different factors. In this study, we explore two potential sources of influences. The first are the three sources of stress in the context of music performance which can affect performance quality (i.e., trait anxiety, situational stress, and task mastery). The second is the listeners’ musical background.

Given these antecedents, we investigate two research questions (RQs):

RQ1: What is the effect of MPA, situational stress, and task mastery on the listener’s perception of expressiveness, while considering their musical background?

Hypothesis 1: There will be differences in perceived expressiveness of the musicians depending on their MPA, situational stress, and task mastery (Wilson and Roland, 2002), when considering the listeners’ musical background.

RQ2: What is the effect of MPA, situational stress, and task mastery on the listener’s interpersonal accuracy, while considering their musical background?

Hypothesis 2: There will be differences in the listeners’ interpersonal accuracy of expressiveness depending on the musician’s MPA, situational stress, and task mastery, when considering the listeners’ musical background (Chang-Arana et al., 2022).

## 2. Methods

### 2.1. Ethical approval

This study was approved by Aalto University Research Ethics Committee and the University of Arts Helsinki – Sibelius Academy.

### 2.2. Stimuli creation procedure

Ten pianists from a leading tertiary music institution in Finland took part in the study (mean age = 23, *SD* = 2.31). The pianists were compensated with 2 credit points, 100 euros (€), and recordings of their performances. They were assured to be compensated even if they would prefer not to share their musical performances after the recitals and withdraw their recordings. We chose pianists because solo instrumentalists may show more MPA than orchestral instrumentalists, particularly as they approach the end of their studies (Casanova et al., 2018; Chang-Arana et al., 2022).

Pianists were initially informed that the purpose of the study was to investigate how the COVID pandemic impacted interactions between musicians and audiences. Since this was a MPA study, it was important not to reveal the true goal of the study. The pianists were contacted 7 weeks before the performing days. They were asked to prepare a programme of two pieces which had to be memorized: one familiar and another one unfamiliar. We defined the familiar piece as one which “you have played it for an audience before,” whereas the unfamiliar piece as “a piece new to you which you have never played for yourself or for anybody else.” After the initial meeting, they completed sociodemographic questionnaires as well as the Revised Kenny-Music Performance Anxiety Inventory (K-MPAI, Kenny, 2009), and other questionnaires not reported here. To control for familiarity with the piece, we only allowed the pianists to start practicing them 3 weeks before the performances, using the cover story that the criteria to choose the unfamiliar piece was still undecided. We instructed the pianists to “choose a piece which you have never played even for yourself and which you think you can get memorized for May’s recital. Remember that this piece should match your current performing level.” To keep a track of their performance practice, we asked the pianists to fill in an online performance diary every time they had a practice session.

Two online recitals were organized and advertised through social media and from mouth-to-mouth 3 days before the first performing day. The pianists were randomly assigned to either day, as well as the order on which they would perform their chosen pieces. The performances took place in the same hall using a Steinway & Sons model C grand piano. The pianists performed their programme twice during their assigned day, first the rehearsal condition and then the online-streaming condition. We chose this order because in real circumstances musicians will have a dress-rehearsal session prior to the actual performance. Here we decided to reproduce that context even though a learning effect could have been introduced. The pianists were instructed to wear the same cloths for both performances. Before starting the rehearsal condition, the pianists were allowed to warm up, complete the State subscale of the State-Trait Anxiety Inventory (STAI, Spielberger et al., 1983), and do a sound check. The State subscale was administered to measure their anxiety before the rehearsal. The pianists were then read the following instructions by Kwan (2016, p. 22):

“You will have 60 min to play your music as many times as you want to until you feel satisfied with the performance, and you can restart the piece at any moment you want, as long as there is a completed performance by the end of the session. You are allowed to take breaks and evaluate your own recordings between performances.”

During the concert condition, the pianists arrived 30 min before the beginning of the concert and completed the State-STAI once again to measure their anxiety before the online performance. They waited on the hallway and came to play one at the time. The only difference from the rehearsal condition was a phone streaming the performance and a laptop connected to Zoom which displayed the audience’s profiles to the pianists. The first author was present with them in both performing conditions.

Each pianist completed a self-rating task based on their own performances. They watched back to approx. 1 min clips of their rehearsal and concert performances and rated each of them according to their expressiveness (Kendall and Carterette, 1990, p. 156; Kwan, 2016) using a 1–100 continuous scale (Chang-Arana et al., 2022):

• How expressive was the rendition of this piece? Musical expression can be likened to the expression of an actor in speaking their part: They may speak in a monotone, in a manner appropriate to the idea, or they might exaggerate.

Then, the pianists were fully debriefed about the objectives of the study as well as the full details of the study design and procedure. During the debriefing we corroborated that none of the pianists guessed that MPA was the real object of study. The clips belonging to pianists with the lowest, middle, and highest scores in the K-MPAI were chosen for the perceptual study (i.e., pianist 1, 5, and 10).

As in Chang-Arana et al. (2022), we will recruit professional pianists with extensive piano performance and teaching experience to watch all clips in counterbalanced order. Using a 10-point Likert scale, the pianists will rate how much did the observed pianists move after watching each clip. The inter-rater reliability of the judges will be calculated through intraclass correlation (ICC, Koo and Mae, 2016).

### 2.3. Questionnaires and materials

#### 2.3.1. Music performance anxiety

The K-MPAI (Kenny, 2009) is a 40-item self-report scale. It was designed by Kenny (2009) after Barlow’s (2000) triple vulnerability model. It explains the origin of anxiety disorders as a consequence of an interaction between three vulnerabilities: biological (hereditary anxiety components), psychological (early experiences resulting in a sense of uncontrollability), and specific life conditioning events. The questionnaire has been translated to different languages such as Portuguese (Rocha et al., 2011; Barbar et al., 2014a,b,c, 2015), Spanish (Zarza-Alzugaray et al., 2016b; Chang-Arana et al., 2018), Romanian (Faur et al., 2021), among others. Furthermore, its psychometric properties have been tested cross-culturally (Chang-Arana et al., 2018). The K-MPAI has shown a strong correlation of *r* = 0.70 with trait anxiety (Chang-Arana et al., 2018).

#### 2.3.2. State-trait anxiety

The STAI (Spielberger et al., 1983) is a 40-items self-report scale. The state and trait subscales contain 20 items each to be rated on a 4-points Likert scale. The internal consistency of the scale ranges from 0.86 to 0.95 (American Psychological Association, 2011), both values above the Nunnally (1987) criterion of 0.70.

#### 2.3.3. Perceived expressiveness

This scale is designed based on Kwan (2016) and defined according to Kendall and Carterette (1990).

#### 2.3.4. Recording equipment

Performances were recorded using Rode NT5 Condenser microphones, a MOTU Ultralite mk4 USB Audio Interface, and a Sony HDR-CV560VE Camcorder.

### 2.4. Perceptual study procedure

One hundred twenty online participants will be recruited using Prolific (Peer et al., 2017), and social media to complete the study. The instructions and tasks will be based on Chang-Arana et al. (2022): Participants will be presented with approximately 1 min clips of each piece (familiar and unfamiliar), performed on both conditions (rehearsal and recital). Clips were edited and the pianists’ faces blurred using Shotcut (Meltytech). Each piece performed in rehearsal and recital condition will be grouped together and presented in random order. After each clip, participants will rate the pianists’ expressiveness as defined in Section “Stimuli creation procedure.” Participants will be asked to self-identify as non-musicians or music-loving non-musician (<6 years of private lessons and <6 years of daily practice and not enrolled in a college music course), amateur or serious amateur musicians (between 6 and 10 years of private lessons and >6 years of daily practice and enrolled in 1–2 non-major music courses), or semi-/professional musicians (>10 years of private lessons and >6 years of daily practice and enrolled in a Bachelor of Music degree), with the question “which title best describes you?” (Zhang and Schubert, 2019). The study was implemented online in Gorilla platform (Anwyl-Irvine et al., 2020).

### 2.5. Data analysis

To answer RQ1, we will conduct a 2 (rehearsal vs. recital) × 2 (familiar vs. unfamiliar) × 3 (low MPA vs. mid MPA vs. high MPA) mixed repeated-measures ANOVA, with musical background (non-musicians vs. amateur musicians vs. semi/professional musicians) as between-subjects variable, and the listeners’ perceived expressiveness scores as dependent variable. To answer RQ2, we will conduct the same analyses, only that the dependent variable will be the listeners’ interpersonal accuracy of expressiveness. For RQ1 and RQ2, we will set our *p*-value to 0.025 (0.05/2 tests conducted with the same data) (Field, 2009). Interpersonal accuracy is defined as the pianist’s self-reported expressiveness on a given clip minus the listener’s perceived expressiveness on the same clip. Our *a priori* repeated measures, within-between interaction calculation of sample size suggests 108 participants, given an effect size *f* = 0.10, α = 0.025, 1-β = 0.80, number of groups = 3, number of measurements = 12, correlation among repeated measures = 0.50, and non-sphericity correction ε = 1 (Faul et al., 2007). Each level of our between-subjects variables will have the same number of participants. We will add equally to each group at least 10% more participants to account for missing values or data points which could be eliminated for justified reasons (e.g., outliers, participants not answering diligently, etc.). Thus, each group will have at least 40 participants.

## 3. Preliminary results

The K-MPAI, Trait-STAI, and State-STAI scores are displayed in Table 1. Table 1 reveals two interesting results. The K-MPAI and Trait-STAI values showed a significant and strong correlation, *r* = 0.75, *p* = 0.013, 95% CI (0.22, 0.94). The strong correlation between the K-MPAI and the Trait-STAI supports (a) our decision to use the scores of the K-MPAI as a measure of trait anxiety and (b) our reasoning to choose pianists 1, 5, and 10. Second, the difference in state anxiety experienced by the pianists before the rehearsal (*M* = 37.40, *SD* = 5.99) and recital conditions (*M* = 38.10, *SD* = 9.17) was not significantly different, even though we would have expected to see higher scores in the recital condition, *t*(9) = −0.44, one-sided *p* = 0.336, 95% CI (−4.32, 2.92). Based solely on the state anxiety scores, listeners may not be able to perceive differences in expressiveness according to performing context.

**TABLE 1:** Pianists’ kenny-music performance anxiety inventory (K-MPAI) and STAI scores.

Pianist	K-MPAI	Trait-STAI	State-STAI		
			Rehearsal	Recital	Difference
1	44	29	37	38	−1
2	44	36	38	28	10
3	60	43	39	40	−1
4	91	48	43	50	−7
5	94	51	36	31	5
6	105	48	37	41	−4
7	106	54	23	22	1
8	107	38	39	43	−4
9	135	52	46	51	−5
10	144	50	36	37	−1

On average, the pianists self-rated their performances during the rehearsal condition as more expressive than in the rehearsal condition (see Table 2 for further details). When performing the familiar pieces in the rehearsal condition (*M* = 79.00, *SD* = 12.67), pianists self-reported more expressiveness than when performing the familiar pieces in the recital condition (*M* = 75.00, *SD* = 14.70). Similarly, pianists self-reported more expressiveness when performing the unfamiliar pieces in the rehearsal condition (*M* = 76.70, *SD* = 8.95) than in the recital condition (*M* = 70.10, *SD* = 16.05). Furthermore, the expressiveness scores showed higher variability for the unfamiliar pieces (*SD* = 13.72) than for the familiar pieces (*SD* = 11.45).

**TABLE 2:** Difference in pianist’s self-reported expressiveness, according to task mastery and situational stress.

Pianist	Familiar piece			Unfamiliar piece		
	Rehearsal	Recital	Expressiveness difference	Rehearsal	Recital	Expressiveness difference
1	95	91	4	85	76	9
2	100	90	10	90	100	−10
3	77	82	−5	65	62	3
4	67	45	22	70	70	0
5	65	60	5	80	65	15
6	81	75	6	86	60	26
7	70	90	−20	78	95	−17
8	90	75	15	75	65	10
9	80	75	5	75	50	25
10	65	67	−2	63	58	5

Next, we focus further into the three pianists chosen for the perceptual study. Following the same procedure described in Chang-Arana et al. (2022), we extracted four acoustic features (duration, tempo, pulse clarity, and intensity) from the recorded pieces using the MATLAB (2021) based MIRtoolbox (Lartillot and Toiviainen, 2007): Duration, tempo, pulse clarity, and intensity (attack leap) (Table 3). Duration was estimated in seconds by dividing the “total samples of each excerpt with the sampling rate (44 kHz)” (Chang-Arana et al., 2022, p. 5). Tempo was obtained with *mirtempo* function (Lartillot, 2021), pulse clarity was detected using the *mirpulseclarity* function (Lartillot et al., 2008), and intensity was calculated using the *mirattackleap* function (Lartillot, 2021). All three pianists played their familiar pieces faster in the recital condition. Regarding the unfamiliar pieces, only Pianist 1 played their selected piece slower. Pianist 10 showed the highest increase in tempo (and less duration) of their selected unfamiliar piece. See Table 3. Next, we present preliminary results with a sample of 30 participants (professional/semi-professional musicians = 10, amateur musicians = 10, non-musicians = 10).

**TABLE 3:** Extracted musical features of pieces performed.

Pianist			Duration (seconds)	Tempo (bpm)	Pulse clarity	Attack leap
1 (Low MPA)	Familiar	Practice	73.24	80.76	0.17	0.12
		Recital	71.29	104.42	0.15	0.15
	Unfamiliar	Practice	63.48	101.60	0.15	0.11
		Recital	64.67	87.28	0.16	0.12
5 (Middle MPA)	Familiar	Practice	77.11	121.39	0.22	0.12
		Recital	76.12	121.00	0.19	0.12
	Unfamiliar	Practice	63.88	136.98	0.27	0.23
		Recital	61.00	138.56	0.25	0.23
10 (High MPA)	Familiar	Practice	98.55	83.76	0.20	0.31
		Recital	97.45	98.26	0.19	0.31
	Unfamiliar	Practice	60.19	146.61	0.22	0.27
		Recital	56.17	151.42	0.20	0.22

### 3.1. RQ1: What is the effect of MPA, situational stress, and task mastery on the listener’s perception of expressiveness, while considering their musical background?

Mauchly’s test suggests that the assumption of sphericity has been met for the MPA levels, χ^2^(2) = 4.74, *p* = 0.094. A significant effect of MPA levels on the perception of expressiveness was observed, *F*(2, 54) = 30.74, *p* < 0.001, η_*p*_^2^ = 0.53. The pianist with the lowest self-reported MPA [*M* = 75.69, *SE* = 1.89, 97.5% CI (71.20, 80.19)] was rated with the highest expressiveness scores, followed by the pianist with the highest self-reported MPA [*M* = 66.75, *SE* = 2.66, 97.5% CI (60.44, 73.06)], and lastly the pianist with the mid self-reported MPA [*M* = 56.61, *SE* = 2.76, 97.5% CI (50.05, 63.16)].

There was a significant effect of familiarity on the perception of expressiveness, *F*(1, 27) = 24.95, *p* < 0.001, η_*p*_^2^ = 0.48. Listeners rated the familiar pieces as more expressive [*M* = 70.93, *SE* = 2.12, 97.5% CI (65.90, 75.96)] than the unfamiliar pieces [*M* = 61.77, *SE* = 2.33, 97.5% CI (56.25, 67.30)].

**FIGURE 1:**
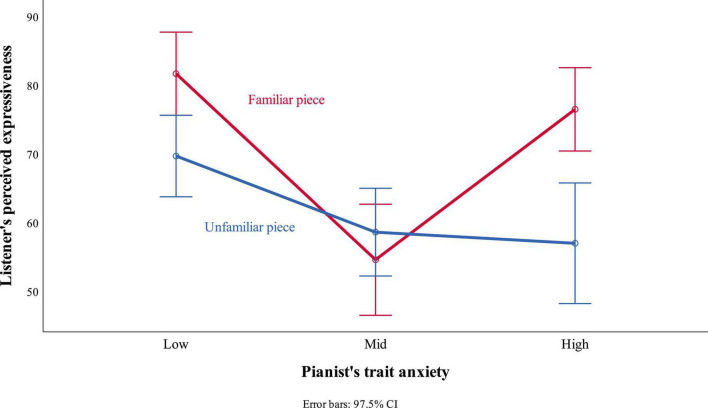
Interaction between self-reported music performance anxiety (MPA) and familiarity with the piece.

Furthermore, a significant interaction between self-reported MPA and familiarity with the piece was observed (Figure 1), *F*(2, 54) = 14.08, *p* < 0.001, η_*p*_^2^ = 0.34. When rating the pianist with the lowest MPA, the unfamiliar piece [*M* = 69.70, *SE* = 2.50, 97.5% CI (63.77, 75.63)] was perceived as less expressive than the familiar piece [*M* = 81.68, *SE* = 2.55, 97.5% CI (75.63, 87.74)]. When rating the pianist with mid MPA, the unfamiliar piece [*M* = 58.62, *SE* = 2.69, 97.5% CI (52.23, 65.00)] was perceived as more expressive than the familiar piece [*M* = 54.60, *SE* = 3.41, 97.5% CI (46.51, 62.69)]. When rating the pianist with high MPA, the unfamiliar piece [*M* = 57.00, *SE* = 3.70, 97.5% CI (48.22, 65.78)] was perceived as less expressive than the familiar piece [*M* = 76.50, *SE* = 2.56, 97.5% CI (70.43, 82.57)].

### 3.2. RQ2: What is the effect of MPA, situational stress, and task mastery on the listener’s interpersonal accuracy, while considering their musical background?

In Table 4, we present a summary of the listeners’ mean perceived expressiveness and the pianists’ self-reported expressiveness. In 9 out of 12 videos, we observe a negative difference. This indicates that, across stimuli, the listeners perceived less expressiveness than what the pianists self-reported.

**TABLE 4:** Listeners’ mean perceived expressiveness and pianists’ self-reported expressiveness.

Trait anxiety	Familiarity and performance condition	Listeners’ mean perceived expressiveness	Pianist’s self-rated expressiveness	Difference
Low	Familiar rehearsal	82.00	95.00	13.00
	Familiar recital	81.37	91.00	−9.63
	Unfamiliar rehearsal	69.93	85.00	−15.07
	Unfamiliar recital	69.47	76.00	−6.53
Mid	Familiar rehearsal	53.40	80.00	−26.60
	Familiar recital	55.80	65.00	−9.20
	Unfamiliar rehearsal	58.87	65.00	−6.13
	Unfamiliar recital	58.37	60.00	−1.63
High	Familiar rehearsal	76.60	65.00	11.60
	Familiar recital	76.40	67.00	9.40
	Unfamiliar rehearsal	57.93	63.00	−5.07
	Unfamiliar recital	56.07	58.00	−1.93

Following a past procedure (Chang-Arana et al., 2022, p. 7), we calculated the difference between the listeners’ perceived expressiveness scores and the pianists’ self-reported expressiveness. This difference was squared and then squared rooted to transform the scores into positive values. Values closer to 0 indicate higher accuracy. Conversely, values larger than 0 indicate lower accuracy.

A significant effect of performance context on the listeners’ accurate inference of expressiveness was observed, *F*(1, 27) = 11.83, *p* = 0.002, η_p_^2^ = 0.31. Listeners were more accurate when inferring the pianists’ self-reported expressiveness when rating the recital condition [*M* = 14.07, *SE* = 1.00, 97.5% CI (11.70, 16.44)] than when rating the rehearsal condition [*M* = 18.54, *SE* = 1.32, 97.5% CI (15.41, 21.68)].

## 4. Preliminary discussion

The pianists’ data suggests that they experienced approximately the same state anxiety before the rehearsal and before the recital. Thus, our expectation of observing higher state anxiety scores in the recital condition when compared to the rehearsal condition was not met.

Yet, it was interesting to observe that the pianists rated their rehearsal performances as more expressive than the recital performances, independently of the familiarity with the piece. In addition, we observed that the pianists with the lowest, middle, and highest MPA performed at a faster tempo in the recital when compared to the rehearsal, independently of the familiarity with the piece (except for the pianist with the lowest trait anxiety who played their unfamiliar piece slower). Taking the observed differences of expressiveness and tempo together, the manipulation of anxiety may have had an effect undetected by the State-STAI. In a previous study, we documented increases in tempo when performing in a recital condition in comparison to a rehearsal condition (Chang-Arana et al., 2022). We drew a parallel of these results to fast speech during public speaking observed in individuals with panic disorder and social phobia (Hagenaars and van Minnen, 2005; Laukka et al., 2008; Chang-Arana et al., 2022).

Preliminary results suggest that the performances of the pianists with the lowest and highest self-reported MPA obtained the highest perceived expressiveness scores, while the pianist with the mid self-report MPA received the lowest perceived expressiveness scores. Although it is soon to confirm this trend, it could be explained by the body movements displayed by the pianists. To control for the known effects of ancillary gestures in the listeners’ heightened perception of expressiveness (Davidson, 1993; Vuoskoski et al., 2014), a group of professional pianists will rate the performers’ body movements (Chang-Arana et al., 2022).

Regardless of the listener’s musical background, they perceived the familiar pieces as more expressive than the unfamiliar pieces. Previous literature has linked inadequate preparation and low self-efficacy of musicians to experiencing higher MPA (Roland, 1994; Kenny et al., 2011, 2012; Biasutti and Concina, 2014; Zarza-Alzugaray et al., 2016a; Casanova et al., 2018). If our future analysis supports our preliminary results, then differences on the preparation for a performance are noticeable to listeners with varied musical experience too.

Preliminary results seem to indicate that the listeners’ inference of expressiveness was more accurate in the recital condition in comparison to the rehearsal condition. Yet, this is not explained by the listener’s IA skills, rather by the pianists’ expressiveness scores approaching the listener’s scores. Pianists reported less expressiveness in the recital conditions in contrast to the rehearsal condition. Table 3 shows that the listeners’ mean perceived expressiveness is similar between performing contexts. Thus, the difference in IA can be attributed to the pianists’ self-rating scores, rather than the listener’s IA skills.

In sum, this study investigates the effect of musicians’ MPA, situational stress, and task mastery on the listeners’ perception of expressiveness and interpersonal accuracy, while considering their musical background. We investigate this through an experimental manipulation where pianists with the lowest, mid, and highest self-reported MPA performed a familiar and an unfamiliar piece in front of an online audience and in absence of an audience. Listeners will be asked to rate the expressiveness of these performances, being blind to the experimental manipulations. The listeners’ IA will be calculated as is the difference between their perceived expressiveness and the pianists’ self-reported expressiveness.

## Data availability statement

The raw data supporting the conclusions of this article will be made available by the authors while complying with GDPR regulations.

## Ethics statement

The studies involving human participants were reviewed and approved by Aalto University Research Ethics Committee. The patients/participants provided their written informed consent to participate in this study.

## Author contributions

ÁC-A designed the study and wrote the manuscript. AM developed the coding and conducted the music information retrieval analyses. NP enabled contacting the pianists who participated, as well as using the facilities of the music institution. MT provided general supervision of the study. All authors contributed substantially in the writing and preparation of the manuscript.
